# Reflecting biomedical, technological and environmental issues of our modern society. The recent “Forum” section in Poiesis & Praxis

**DOI:** 10.1007/s10202-011-0097-7

**Published:** 2011-10-18

**Authors:** Stephan Lingner

**Affiliations:** Europäische Akademie zur Erforschung von Folgen wissenschaftlich-technischer Entwicklungen Bad Neuenahr-Ahrweiler GmbH, Wilhelmstr. 56, 53474 Bad Neuenahr-Ahrweiler, Germany

This issue of Poiesis & Praxis will give—by means of its recent “Forum” section—opportunity to a broader and enhanced debate about new and emerging fields of research, thus contributing to early assessments and ethical evaluation of recent developments in science and technology. Nevertheless, “early warnings” in this field—although favorable—basically suffer from still poor knowledge on corresponding evolving developments and on their chances and risks to the society. The underlying problem is known as the so-called Collinridge-dilemma, which describes the necessity and—at the same time—the difficulty for timely science and technology governance.^1^ This situation leads scholars of science and technology studies to formulate reasonable but yet provisional assessments of respective developing challenges. Therefore and while being most up-to-date, corresponding discussion notes generally cannot be considered as mature and sophisticated as extended original papers dealing with the critical review and reflection of already established fields in science and technology. On the contrary, the provisional nature of some conclusions presented in the papers of this issue is welcome with respect to the aim to stimulate and foster further academic discussions on how to shape our technological future—in the most general sense as well as within upcoming issues of this journal.

This “Forum” section exposes a wide field of technology reflection, ranging from biomedical issues over developments within robotics to the adequate design of climate policies. The article by S. Benöhr-Laqueur on *Fighting in the legal grey area* discusses the problems and needs of an interdisciplinary dialog in the case of pre-implantation diagnostics which normative implications are still ambiguous—at least from a German perspective. Another contribution on *Privacy revisited?* by A. Bialobrzeski reflects different bioethical concepts that might be applied to the adequate arrangement of medical biobank regimes. The main problem within this is based upon the dilemma between social interests in broad access to patient data on one hand and reasonable privacy rights of the individuals on the other hand. The author proposes a contested concept along Rawls’ theories for a just solution of the data handling problem. The paper of M. Decker et al. on *Service robotics* frames an interdisciplinary technology assessment on the changing roles and environments of sophisticated modern robots that will intrude progressively in peoples’ daily life and might affect the autonomy of the human users. The question whether “you know your new companion” is leading for the formulation of a corresponding interdisciplinary research initiative by the authors. Finally, D. Cansier’s article on *Rainforest conservation as a strategy of climate policy* unfolds a promising concept for the still pending implementation of rainforest conservation and utilization as an element of international climate protection policy. He proposes respective emission credits and certificates as instruments for the realization of his ideas and states that the combination of corresponding national and de-central approaches might give the best results for all relevant actors.

The quoted papers resemble the full spectrum of technology assessment. Those readers who feel addressed or even challenged by the articles of this issue are highly welcome to bring in their points of view and to debate the given theses as well as to elaborate new discussion notes on other emerging themes by submitting their papers to Poiesis & Praxis. In this way, the journal might even literally serve as a *forum* of interdisciplinary communication and exchange of ideas. Therefore, the editors of this journal are curious about the corresponding feedbacks.

## Footnotes

1 See Collingridge (1980).
